# *Holothuria Leucospilota* Polysaccharides Ameliorate Hyperlipidemia in High-Fat Diet-Induced Rats via Short-Chain Fatty Acids Production and Lipid Metabolism Regulation

**DOI:** 10.3390/ijms20194738

**Published:** 2019-09-24

**Authors:** Yiqiong Yuan, Qibing Liu, Fuqiang Zhao, Jun Cao, Xuanri Shen, Chuan Li

**Affiliations:** 1 Hainan Provincial Engineering Research Centre of Aquatic Resources Efficient Utilization in the South China Sea, Key Laboratory of Marine Food Processing of Haikou, College of Food Science and Engineering, Hainan University, Haikou 570228, China; yiqiongyuan@126.com (Y.Y.); zhaoqiang9926@163.com (F.Z.); shenxuanri2009@163.com (X.S.); 2 Department of Pharmacology, School of Basic Medicine and Life Science, Hainan Medical University, Haikou 571199, China; Qibing.liu@hainmc.edu.cn; 3 Collaborative Innovation Center of Seafood Deep Processing, Dalian Polytechnic University, Dalian 116034, China

**Keywords:** *Holothuria leucospilota*, polysaccharides, hyperlipidemia, high-fat diet, short-chain fatty acids, lipid metabolism, gut microbiota

## Abstract

*Holothuria leucospilota* polysaccharides (HLP) are expected to become potential resources for the treatment of hyperlipidemia because of their various bioactivities. In the study, the treatment of HLP on improving hyperlipidemia in rats was explored. Oral administration of HLP at 100 or 200 mg/kg body weight effectively alleviated serum lipid levels and liver histological abnormalities in high-fat-diet rats. HLP regulated abnormal mRNA, lipogenesis-related hormones and inflammatory cytokines (tumor necrosis factor-α, interleukin-6 and interleukin-12) levels. HLP improved the ability of gut microbiota to produce short-chain fatty acids (SCFAs). SCFAs have been found to ameliorate liver lesions. Therefore, HLP alleviated hyperlipidemia by improving the levels of SCFAs to regulate lipid metabolism. These results indicated that HLP could be used as beneficial polysaccharides to alleviate hyperlipidemia.

## 1. Introduction

Hyperlipidemia, a common metabolic syndrome worldwide, is one of the leading risk factors of vascular disease. Generally, hyperlipidemia is clinically manifested by elevated levels of total cholesterol (TC), triglyceride (TG) and low-density lipoprotein cholesterol (LDL-C) [1,2]. Therefore, a reduction in serum lipid levels is a critical approach which is used to prevent or retard the formation of hyperlipidemia. Moreover, hyperlipidemia is connected with the induction of inflammation and some abnormal adipokines (such as insulin, adiponectin and leptin) production. An unbalanced diet is considered as one of the reasons for hyperlipidemia. Long-term high-fat-diet intake can alter the overall structure of the gut microbiota, leading to inflammation and insulin resistance [3,4]. A high-fat diet is predominantly metabolized in the liver by accelerating de novo lipogenesis and increasing very-low-density lipoprotein biogenesis. High-fat diets have been shown to cause oxidative stress by increasing reactive oxygen species and reducing antioxidant enzymes. It was found to be associated with over expression of a wide variety of inflammatory and metabolic disease states [5]. As the intake of high-fat food has increased dramatically, hyperlipidemia has become the cause of low-quality life and substantial economic burden globally. Although there was some progress in developing drugs to treat hyperlipidemia, the side effects of conventional drugs are emerging as time goes on [6]. Therefore, considerable efforts have been devoted to developing new sources from natural foods for alleviating hyperlipidemia.

Sea cucumber has been a traditional tonic food in China and some Southeast Asia countries for thousands of years [7]. The body wall of sea cucumber contains various active components, such as polysaccharides, collagens, and saponins. Polysaccharides are the most important ingredient of sea cucumber due to multiple bioactivities. Accumulating evidence has demonstrated that polysaccharides extracted from sea cucumber showed a wide range of pharmacological effects, including anti-obesity, improving glucose tolerance and being cardio-protective [8,9,10,11]. Fucoidan from *Isostichopus badionotus* improved insulin resistance and inflammatory response. Hang et al. demonstrated that sea cucumber polysaccharides were natural antioxidants that could be utilized as a therapeutic supplement for dyslipidemia from diet intervention [12]. Sulfated polysaccharides from sea cucumber have also been found to have a good effect on reversing hyperlipidemia [13]. Thus, the biological activities of sea cucumber polysaccharides have attracted wide attention.

Studies showed that the health-promoting potential mechanisms of sea cucumber polysaccharides are related to various pathways. For instance, sea cucumber polysaccharides enhance insulin sensitivity [14]. Inflammation is also considered as one of the most innovative concepts in the mechanism. In addition, sea cucumber polysaccharides improve the serum lipid level through the regulation of the expression of lipogenesis-related genes and the ability of gut microbiota to produce SCFAs, thereby improving gut health [15]. Notably, SCFAs play an essential part in the energy source for some bacteria and gut epithelial cells as well as ameliorate serum cholesterol levels. SCFAs have aroused great attention in the prevention of various diseases.

Polysaccharides from *Holothuria leucospilota* (HLP) were isolated as a previous study [16]. Moreover, it has been exhibited that the mucopolysaccharides from *Holothuria leucospilota* have potential therapeutic application as antitumor [17]. Some previous reports showed that HLP have the property of hyperlipidemia alleviation, but the mechanism has not been fully understood yet. Thus, the treatment of hyperlipidemia by HLP was explored in this study. The aim of the result is to provide a new source of functional food to alleviate hyperlipidemia.

## 2. Results and Discussion

### 2.1. The Effects of HLP on Normalizing Serum Lipid Metabolic Disorders

**Figure 1:**
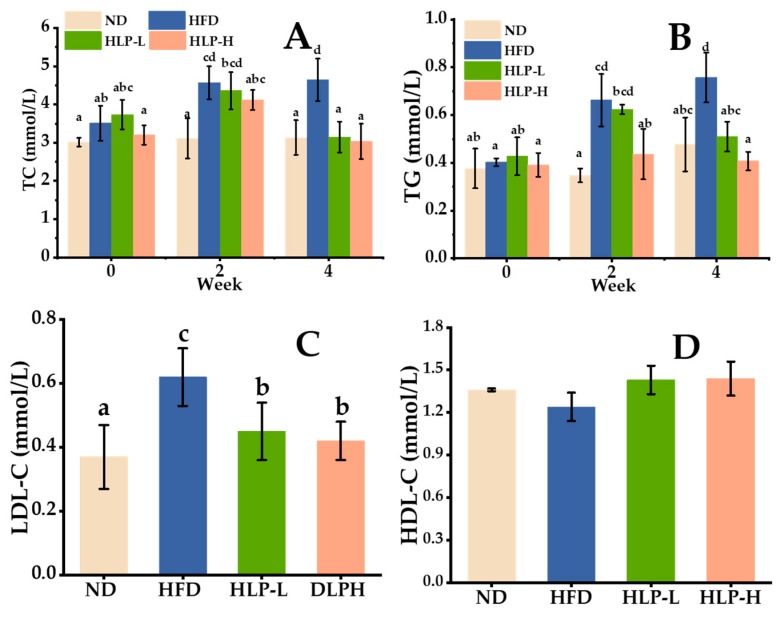
Effect of *Holothuria leucospilota* polysaccharides (HLP) on serum total cholesterol (TC) (**A**), triglyceride (TG) (**B**), low-density lipoprotein cholesterol (LDL-C) (**C**) and high-density lipoprotein cholesterol (HDL-C) (**D**). Data are presented as means ± standard error of the mean (SEM) (*n* = 8); values with different superscripts represent significant differences (*p* < 0.05).

TC and TG levels are essential indicators of hyperlipidemia [18]. Figure 1A,B) showed that the TC (approximately 3 mmol/L) and TG (approximately 0.38 mmol/L) levels remained stable under normal diet (ND, the control group) during the four weeks. While the serum TC and TG levels of high-fat diet (HFD, the model group) dramatically increased in the second week (4.5 and 0.65 mmol/L), then up to 4.8 and 0.75 mmol/L (*p* < 0.05) in the fourth week. Compared with the HFD, HLP-H (the group of high dose sea cucumber polysaccharides) prominently decreased TC and TG levels (12% and 40%, respectively) in the second week. HLP-L (the group of low dose sea cucumber polysaccharides) also showed the same effects in the fourth week. The result was similar to that of some highly-purified natural polysaccharides, where TG levels were decreased after a 10-day treatment of β-glucan (50 mg/kg) [19]. In addition, high-density lipoprotein cholesterol (HDL-C) was thought to accelerate lipids migration from peripheral tissues to the liver. Excessive LDL-C was oxidized to toxic ox-LDL leading to a series of metabolic diseases [20]. The final LDL-C and HDL-C levels were measured in the fourth week (Figure 1C,D). Compared with ND, HFD resulted in a significant increase in the LDL-C level (0.62 mmol/L, *p* < 0.05). Whereas, oral administration with HLP reversed this tendency, showing a distinctly lower LDL-C level than that of the HFD (approximately 0.41 mmol/L, *p* < 0.05). The level of HDL-C in all groups was stable at approximately 0.5 mmol/L and fluctuated slightly. Some natural polysaccharides appeared to have an effective lipid-lowering function. Mannan, a natural biological macromolecule of polysaccharide, decreased TC, LDL-C, and TG at the dose of 50 mg/kg, but, at that dose of mannan, did not normalize serum lipids to those values determined for control levels [21]. The results revealed that the high-fat diet caused a lipid metabolism disorder, and HLP showed a positive effect on serum lipid profile levels. As active compounds, sea cucumber polysaccharides, such as *Isostichopus badionotus* [9], *Pearsonothuria graeffe* and *Isostichopus badionotus* [20] have also been reported to offer potent functions in improving abnormal serum lipids levels.

### 2.2. HLP Alleviated the Hormone Levels in Hyperlipidemia Rats

**Figure 2:**
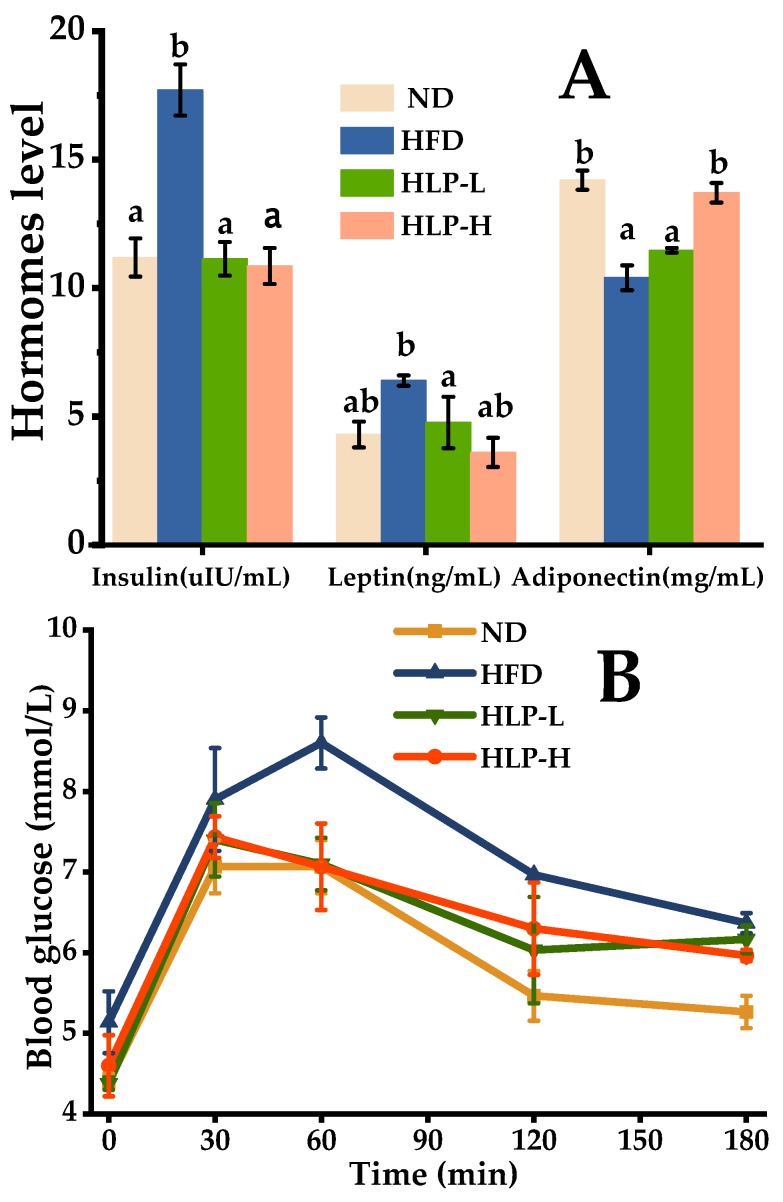
Effects of HLP on adiponectin, insulin, and leptin level (**A**); glucose response curve during the insulin tolerance test (**B**). Data are presented as means ± SEM (*n* = 8). Values with different superscripts represent significant differences (*p* < 0.05).

Insulin is known as the most effective synthetic metabolic hormone [22]. The evidence suggested that hyperlipidemia was mainly driven by insulin resistance [23,24], which was an aggregation of related with metabolic abnormalities of hyperlipidemia, glucose intolerance, and the enhancing of LDL-C [22]. In this study, the serum insulin concentration of rats in HFD (17.7 μIU/mL, *p* < 0.05) was significantly higher than that of ND (11.2 μIU/mL, Figure 2A). Less effective glucose clearance after glucose administration was also observed in the HFD (Figure 2B). Blood glucose levels in the HFD were higher than the ND at 60 min, 120 min, and 180 min. It revealed that insulin resistance had formed in the rats treated with the high-fat diet and this result was consistent with other reports [25]. Polysaccharides prevent the development of hyperglycemia by increasing glucose tolerance and insulin sensitivity [11]. HLP treatment effectively inhibited insulin resistance and enhanced glucose clearance. The insulin concentrations tended to normal levels and glucose clearance was significantly higher at 60 min.

The function of leptin is regulating energy expenditure and appetite, as well as stimulate macrophages to secrete TNF-α, IL-6, and IL-12. A prominent elevation of leptin level was detected in the HFD. The situation was effectively ameliorated after the therapy of HLP. The leptin content that intervened on HLP-H was 3.70 ng/mL (42.50% lower than HFD). Leptin levels in the normal range is used for inhibiting appetite, enhancing fatty acid oxidation, and reducing body fat. However, excessive levels of circulating leptin can be caused by increasing subcutaneous fat and obesity [26]. Additionally, adiponectin is an endogenous active substance secreted by adipocytes, which regulates glucolipid metabolism and energy homeostasis [26]. The high-fat-diet intake significantly dropped in the adiponectin level (10.4 mg/mL) compared with those of ND (14.2 mg/mL) by 30% (*p* < 0.05). It manifested that hyperlipidemia induced by high-fat-fed rats disrupted the adiponectin level. The decreased level of adiponectin can aggravate insulin resistance [27]. Fortunately, HLP-H feeding results in an amelioration in the level of adiponectin. Additionally, the higher levels of adiponectin inhibit the inflammation via a reduced expression of pro-inflammatory cytokines and enhance the expression of anti-inflammatory cytokine IL-10.

### 2.3. HLP Inhibit Inflammation in Hyperlipidemia Rats

Patients with chronic metabolic disorders often exhibit abnormal levels of many inflammatory cytokines. In the study, the level of tumor necrosis factor-α (TNF-α) in the HFD was increased by 38.8% (Table 1, *p* < 0.05) than that in the ND, whereas treatment with HLP effectively reduced the production of it (*p* < 0.05). TNF-α is mainly produced by macrophages, which can cause vascular damage and promote the release of other inflammatory cytokines [28]. IL-6 promotes vascular disease by up-regulating the ability of macrophages to degrade oxidized-LDL [29,30]. IL-12 promotes differentiation of pro-inflammatory cells and promotes atherosclerosis [30]. Compared with the levels of the ND, the interleukin-6 (IL-6) and interleukin-12 (IL-12) levels in the HFD were markedly increased (36.5% and 42.8%, respectively; *p* < 0.05). Some studies also showed that a high-fat-diet fed for four weeks showed increased inflammatory signaling [31]. Additionally, tissue inflammation was caused by the enhancement of macrophage infiltration to express more inflammatory cytokines. HLP-administered rats had significantly lower production of IL-6 and IL-12, suggesting the anti-inflammatory activities of HLP. Meanwhile, the concentration of IL-10 (a critical anti-inflammatory cytokine) significantly increased (especially in HLP-H, *p* < 0.05). These results implied that the administering of HLP could inhibit the development of inflammation.

### 2.4. The Changes of Gut Microbiota in Hyperlipidemia Rats

**Figure 3:**
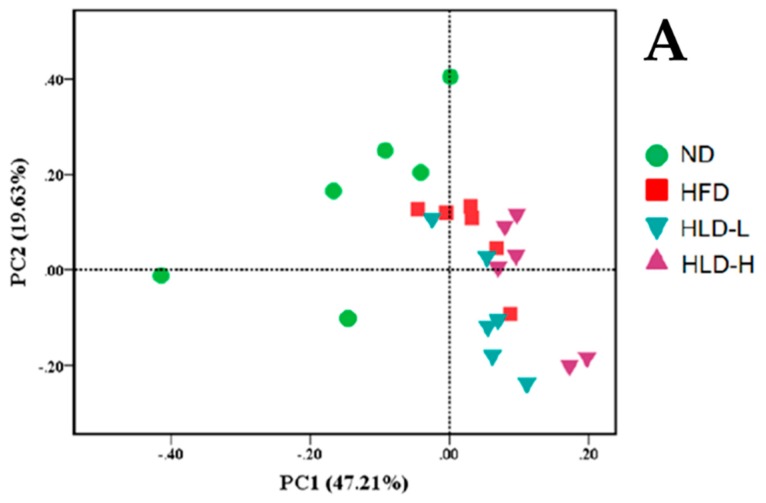

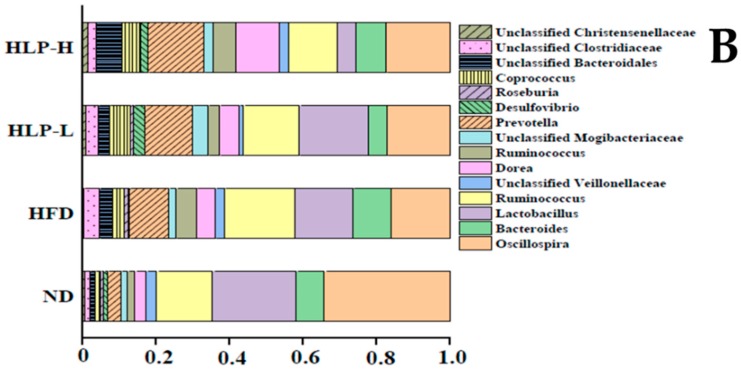
Effects of HLP on the gut microbiota. Principal component analysis (PCA) score plot for the gut microbiota (**A**). The bacterial community at the genus level in the guts of the rats (**B**) (*n* = 6).

The overall structural changes of gut microbiota were also analyzed using principal component analysis (PCA, Figure 3A). It showed that there were differences in the gut microbial composition when treated with a normal diet and a high-fat diet, and diet was a key factor shaping gut microbiota. The changes in gut microbiota with metabolic disorders caused by the high-fat diet are related to the increase of energy harvesting from the diet and the changes in fatty acids metabolism in the liver. Notably, the imbalance was considered to be an essential factor in the pathogenesis of diverse diseases [32]. However, HLP consumption for four weeks did not effectively reverse the genus-level microbial compositions of high-fat-fed rats. The results showed that administration of HLP continually four weeks did not markedly affect the formation of gut microbiota in rats.

By analyzing the composition of the genus level, some differences in the gut microbial composition were observed when treated with a high-fat diet and a normal diet (Figure 3B). The relative abundances of the *Oscillospira* in ND, HFD, HLP-L, and HLP-H were 34%, 16%, 17%, and 17%, respectively. Some studies have certified that *Oscillospira* is related to the leanness of host. In addition, *Oscillospira* probably produces butyric acid, which has a negative correlation to inflammatory diseases [33]. However, HLP treatment did not effectively improve the reduction of beneficial gut microbiota caused by a high-fat diet. Accordingly, the gut microbiota might not be a direct factor in the capacity of HLP to improve hyperlipidemia in the high-fat-diet rats. Similar conclusions have been found in other studies [13].

### 2.5. HLP Altered the SCFA Profile

**Figure 4:**
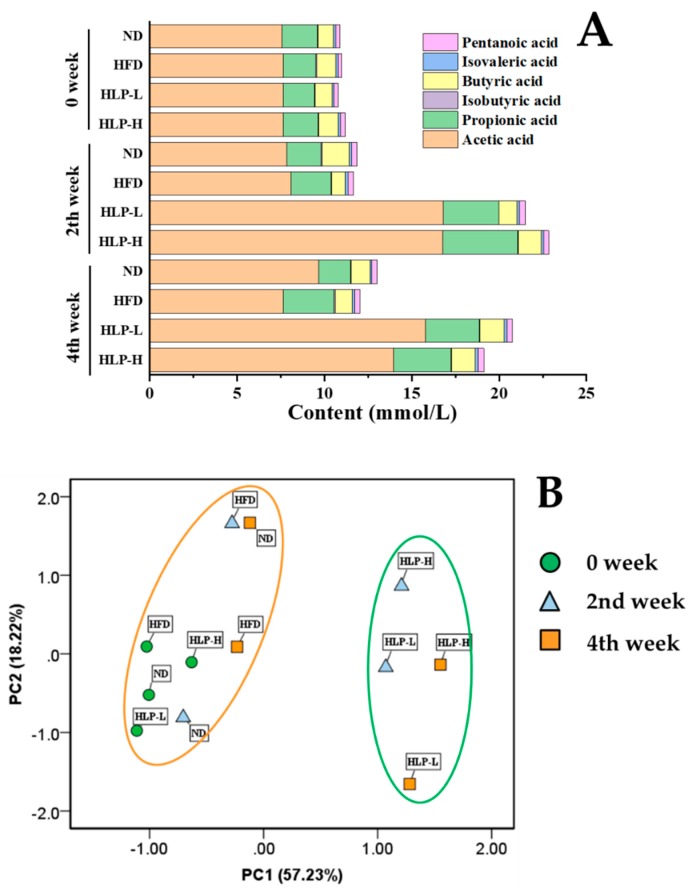
Effects of HLP on short-chain fatty acids (SCFAs). The content of SCFAs in feces (**A**). PCA score plot for SCFAs (**B**) (*n* = 8).

The three dominant SCFAs in feces of rats were acetic, propionic, and butyric acid (Figure 4A). The contents of isovaleric and pentanoic acid were shallow in all the groups. Compared with the ND, SCFAs concentrations of HFD fluctuated slightly but remained relatively stable. However, the administration of HLP for two weeks showed an obvious increase in acetic acid (from 7.6 to 16.8 mmol/L, *p* < 0.05) and the propionic acid contents (from 2.0 to 4.3 mmol/L, *p* < 0.05) in the rat guts. SCFAs participate in lipid metabolism in the liver. Acetic acid is the most abundant substance in intestinal SCFAs and help to down-regulate the expression of ACC and other fat-producing related factors. Propionic acid has a positive effect on cholesterol metabolism. Interestingly, the trend was the same as the changes in TC and TG levels [34,35,36]. In addition, HLP-H displayed a distinct increase in the release of isobutyric acid (approximately 0.09 mmol/L) in the fourth week (*p* < 0.05). The levels of butyric and isovaleric acid in the HFD exhibited the same trend. Overall, compared with the ND and HFD, HLP groups prominently enhanced the content of total SCFAs. Moreover, the score plot of PCA revealed a distinct difference between HLP with ND and HFD (Figure 4B). The levels of SCFAs in ND and HFD were clustered at 0, 2, and 4 weeks, but the HLP-treated groups were clearly distinguished from them in PC1 direction from the second week. It showed that HLP-treatment improved SCFAs contents in the feces of the rats. Based on previous studies, HLP may significantly increase the SCFAs-producing capacity of gut microbiota through the activity of key synthetic enzymes and strengthen gene expression [15].

### 2.6. The Effects of HLP Inhibited Liver Lesions

**Figure 5:**
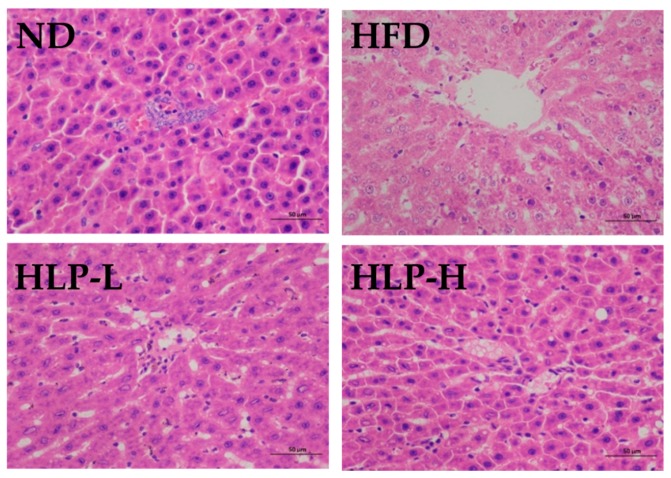
Histological assessment of livers (H&E stain, 200 × magnification).

Pathological characteristics of the liver were investigated to analyze the functions of the HLP on hyperlipidemia. The liver sections of ND were dark reddish and had an intact structure, prominent nucleus, abundant cytoplasm, and visible central vein (Figure 5). High-fat-diet feeding did not caused significant increase of body weight (Appendix A Table A1), but caused morphological tissue degeneration, including fat droplet accumulation, central vein congestion, hepatic cords disorganization, and inflammatory cells infiltration. Liver steatosis occurs when a high-fat diet overloads lipid metabolism in the liver [20]. Inflammatory cells infiltration was observed in liver tissue, which suggested some inflammation had occurred in high-fat-fed rats. HLP supplementation (100 and 200 mg/kg BW) remarkably attenuated the vacuolar degeneration, and only minor inflammatory and fat cells were observed. The phenomenon showed HLP significantly improved liver histological lesions. Many studies also showed that the positive effects of the polysaccharides were linked with reduced fat accumulation in organs [10]. Based on these results, a high-fat diet induced the lesions of liver in rats, while the administration of HLP effectively inhibited these negative effects.

### 2.7. HLP Regulated Lipogenesis-Related Genes in Hyperlipidemia Rats of Liver

**Figure 6:**
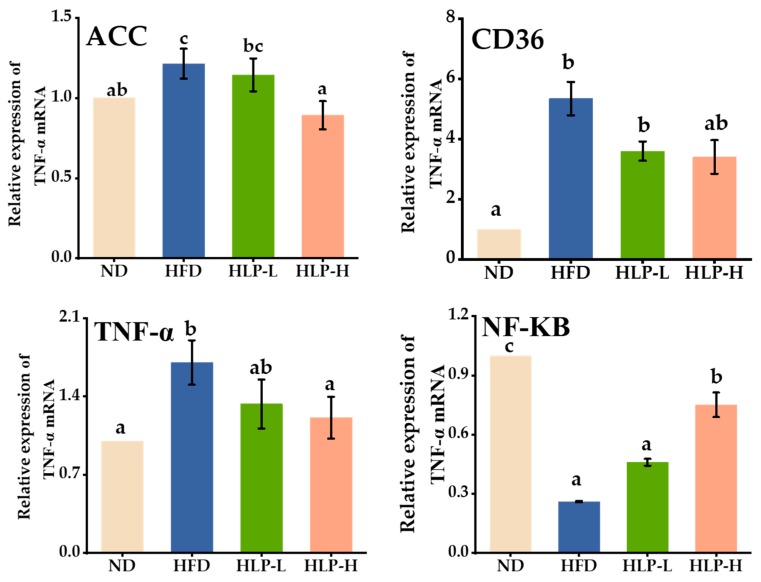
Effects of HLP on the expression of lipogenesis-related mRNA. Data are presented as means ± SEM (*n* = 8). Values with different superscripts represent significant differences (*p* < 0.05).

ACC is a key gene involved in fatty acids synthesis and oxidation [37]. Compared with the ND, the high-fat diet up-regulated the expression of ACC (Figure 6). Activation of ACC increased the level of fatty acids in liver, which further led to pathological changes [38]. However, HLP (200 mg/kg BW) treatment effectively reversed the overexpression of the ACC, which was expected to reduce the synthesis of fatty acids [39]. The CD36 acts as a scavenger receptor, which transfers numerous small biomolecules (such as fatty acids) from serum to cell. The CD36 also binds to ox-LDL and promotes cholesterol accumulation [40]. High-fat-diet feeding remarkably increased the expression of CD36. In contrast, the administration of HLP significantly prevented this adverse change in rats. It indicated that HLP administration reduced the influx of fatty acids into the hepatocyte by decreasing expression of CD36, thereby protecting the liver from further damage [41]. In addition to TNF-α, nuclear factor-kappa B (NF-κB) is also a key gene to regulate inflammation through the transcription and activation of pro-inflammatory cytokines [42]. The HFD had a higher expression of TNF-α (1.7-fold up-regulation) compared with the ND (*p* < 0.05), whereas HLP-H significantly reversed the effects. The expression level of NF-κB in the HFD was prominently down-regulated compared with the level of the ND. Generally, liver lipid accumulation induced by high-fat diet in rats leads to hepatic inflammation by activating NF-κB [43]. Considering that HLP was observed to up-regulate the expression of NF-κB prominently, it can be assumed that HLP is able to inactivate NF-κB and inhibit liver inflammation. These observations suggest that HLP could protect the liver of hyperlipidemia rats.

### 2.8. HLP Alleviate Hyperlipidemia via Enhancing SCFAs to Regulate Lipid Metabolism

**Figure 7:**
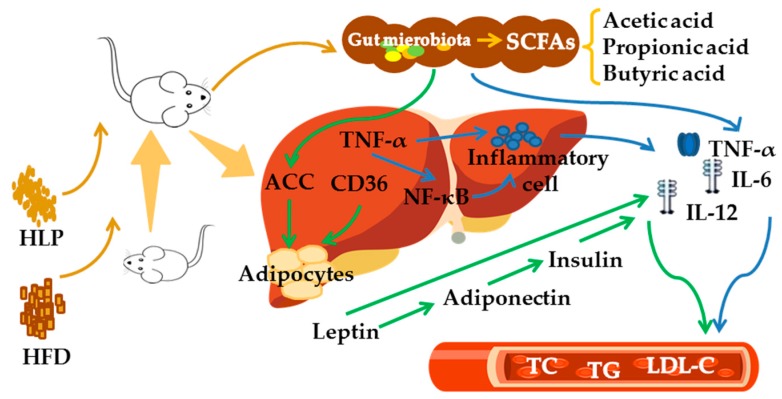
Summary of the mechanism of HLP to prevent hyperlipidemia.

The schematic diagram shows the treatment of HLP alleviates hyperlipidemia induced by high-fat diet (Figure 7). HLP is difficult to digest in the upper gastrointestinal tract. It is transformed into SCFAs by gut microbiota in the large intestine and participates in the regulation of lipid metabolism. HLP suppressed ACC and CD36 expressions to avoid lipid accumulation in liver cells, which inhibited adipocytes from secreting excess leptin and adiponectin. Leptin and adiponectin promoted the secretion of inflammatory factors and increased the level of insulin, which led to insulin resistance. In addition, HLP down-regulated the expression of hepatic inflammation-related genes (TNF-α and NF-ĸB), which also directly inhibited the infiltration of inflammatory cells and alleviated the formation of hyperlipidemia. Therefore, HLP ameliorate hyperlipidemia in high-fat diet-induced rats *via* SCFAs production and lipid metabolism regulation.

## 3. Materials and Methods

### 3.1. Materials

The dry black sea cucumbers (*Holothuria leucospilota*) were obtained from Market Property Development Co., Ltd. (Haikou, China). The dry sea cucumbers were 70–90 mm long and 18–22 g in weight. The species was identified by Prof. Feng Yongqin of Hainan University.

### 3.2. HLP Preparation

Dry sea cucumber powder was obtained by soaking, pulping and freeze-drying. The powder was hydrolyzed with cetylpyridinium chloride and papain to precipitate the sulfated polysaccharides and then dissolved using 4 mol/L NaCl:ethanol (100:15 *v*/*v*). The mixture was kept at 4 °C for 12 h and centrifuged (3500× *g* for 15 min). The sediment was dialyzed with distilled water for 72 h and freeze-dried to obtain HLP [16]. The molecular weight of HLP was 52.80 kDa. The monosaccharides composition were primarily rhamnose, fucose, and glucuronic acid, and the corresponding average mass ratio (*w*/*w*) was 39.08%, 35.72%, and 10.72%, respectively.

### 3.3. Animals and Ethics Statement

Thirty-two male Wistar rats (weighing 200 ± 20 g, four weeks old) were purchased from Tianqin Biotechnology Co., Ltd. (Changsha, China; certificate number: SCXK (Xiang) 2014-0011). The animals were controlled in cages at an ambient temperature of 23 ± 2 °C and a 12:12 h light–dark cycle with free access to water and feed. All procedures were performed following the National Guidelines for Experimental Animal Care and Use, and all efforts were made to minimize suffering. Rat experiments were approved by the Animal Ethics Committee of Hainan Medical University (No. HY-2018-033, 21 Sep 2019), and performed from 1 Nov 2018 to 30 Dec 2018.

### 3.4. Experimental Design

After adaptive feeding for a week, the rats were randomly divided into four groups (*n* = 8). The control group (ND): the rats were fed with a normal diet. The high-fat-diet group (HFD): the rats were fed on a high-fat diet. The components of the high-fat diet consisted of 55% normal diet (22.0% protein + 5.0% fat + 7.0% fiber + 12.2% moisture + 6.0% ash + 1.9% Ca + 0.8% P) + 23% fat + 20% sucrose + 1% cholesterol + 0.2% cholate (Shengming Scientific Animal Farms, Nanjing, China). The low dose polysaccharides group (HLP-L): The rats were fed on the high-fat diet and HLP (100 mg/kg body weight). The high dose polysaccharides group (HLP-H): The rats were fed on the high-fat diet and HLP (200 mg/kg BW). The volume of gavage feeding solution was approximately 1 m per day per rat for each treatment. The ND and HFD were fed with distilled water of the same volume. After four weeks, the rats were starved for 10 h to measure glucose tolerance and blood collected from the orbit. The serum was separated from the blood samples by centrifution (5000× *g* for 10 min), which were used for the following biochemical analysis. Moreover, the rats were executed and dissected after anesthesia (10% chloralic hydras intraperitoneally injected). The liver were harvested and immediately fixed in formalin and RNA fixed until the determination in one month.

### 3.5. Glucose Tolerance Test

Glucose tolerance tests were conducted with a glucose solution (2 g/kg BW). Blood glucose levels (mmol/L) were measured from the tail vein using a touch glucometer (Optium Xceed, Abbot, Berkshire, England) at 0, 30, 60, 90, 120,150, and 180 min after injection.

### 3.6. Serum Biochemical Analysis

Blood samples were collected from the orbit every two weeks during the experiment. The levels of TC, TG, LDL-C, and HDL-C in serum were estimated by corresponding reagent kits (Jiancheng, Nanjing, China). TNF-α, IL-6, IL-12, IL-10, insulin, leptin, and adiponectin were tested by an enzyme-linked immunosorbent assay (ELISA) kits (Sino-UK Institute, Beijing, China).

### 3.7. The Gut Microbiota Analysis by 16S rDNA High-Throughput Sequencing

Total DNA was extracted from microbiota based on a previous study [3]. The V3-V4 region of the bacterial 16S rRNA gene was amplified using a special primer and recovery and purification of amplified products. The quality evaluation was performed by 2% agarose gel electrophoresis. Subsequently, 2 × 300 bp dual terminal sequencing was performed using the Illumina MiSeq platform (Personal Biotechnology, Shanghai, China).

Evaluation and elimination of chimeric sequences used the QIIME v1.8.0 software (Gregory Caporaso, AZ, USA) package. The sequences mentioned above were conducted according to 97% similarity and selected by the operational taxonomic unit (OTU) method. Comparing the OTU representative sequence with the template sequence of the corresponding database (Greengenes, Release 13.8), the taxonomic information corresponding to each OTU was obtained.

### 3.8. SCFAs Analysis

Fecal samples were collected every two weeks from each rat. Faeces SCFAs were detected using a gas chromatography (GC) system as previously reported, with a few modifications [44]. Approximately 0.6 g of the feces sample was weighed and added to 3 mL of water. Then the samples were ground with a glass homogenizer in the ice-cold water and centrifuged at 5000× *g* for 15 min at 4 °C.

The supernatant liquid was analyzed by Agilent 7890A GC equipped with a flame ionization detector (FID) and an HP-FFAP column (30 m × 0.32 mm × 0.25 µm, Agilent Technologies, CA, USA) according to the following program: 80 °C for 0.5 min, 80–150 °C at 4 °C/min and then 150–230 °C at 20 °C/min held for 10 min. The signal was detected at 300 °C with the FID.

### 3.9. Histological Examinations 

Fixed tissues were processed for paraffin embedding. 4 μm slices were prepared and dyed with hematoxylin-eosin. The stained areas were viewed using an optical microscope (Nikon Eclipse CI, Tokyo, Japan) with a magnifying power of × 200.

### 3.10. RNA Preparation and Real-Time PCR

Total RNA was extracted from the liver tissues using the protocol described from the RNA prep Pure Tissue Kit (Tiangen, Beijing, China). Reverse transcription was performed with the First Strand cDNA Synthesis Kit (Fermentas, New York, USA). After five times dilution, 5 μL cDNA product in 25 μL system was used for qPCR amplification. PCR was implemented using SuperReal Preix Plus (SYBR Green) (Tiangen, Beijing, China) by the CFX Connect Real-Time PCR System (Bio-Rad, California, USA). The expressions of mRNA were normalized to β-actin levels, and the calculation method was 2−^△△CT^. The primers used in this study are listed in Appendix B Table A2.

### 3.11. Statistical Analysis

The results were expressed as the mean ± standard error of the mean (SEM) and analyzed by SPSS 24.0 software (SPSS Inc., Chicago, IL, USA). One-way analysis of variance (ANOVA) with Duncan’s multiple range test was used to compare the differences among the various groups. A *p*-value < 0.05 indicated statistical significance.

## 4. Conclusions

It is urgent to find new and safe materials to treat hyperlipidemia. In this study, hyperlipidemia was induced by a high-fat diet in rats, and HLP was used to treat the hyperlipidemic rats. The results showed that HLP alleviated serum lipid levels and histological abnormalities, and enhanced the production of SCFAs in feces. These results suggest the beneficial effect of HLP on hyperlipidemia is through converting HLP into SCFAs by gut microbiota. SCFAs participate in lipid metabolism in the liver. The results provide promising evidence that HLP has potential as a nutraceutical or is potential material for drugs to target hyperlipidemia.

## Figures and Tables

**Table 1:** HLP had different effects on suppressing the chronic inflammation caused by HFD.

Groups	TNF-α (pg/mL)	IL-6 (pg/mL)	IL-10 (pg/mL)	IL-12 (pg/mL)
ND	84.71 ± 1.48 ^b^	151.97 ± 6.73 ^b^	13.22 ± 4.64 ^ab^	24.30 ± 4.79 ^a^
HFD	117.63 ± 19.46 ^c^	207.38 ± 24.50 ^c^	9.65 ± 0.80 ^a^	34.71 ± 3.50 ^b^
HLP-L	48.91 ± 16.11 ^a^	140.44 ± 7.67 ^b^	15.97 ± 3.81 ^ab^	25.32 ± 4.40 ^a^
HLP-H	42.82 ± 7.49 ^a^	138.05 ± 20.72 ^a^	17.49 ± 3.85 ^b^	21.89 ± 6.66 ^a^

Data are expressed as the mean ± SEM (*n* = 8). Values within a column with different letters are significantly different (*p* < 0.05)

## Appendix A

**Table A1:** The sequences of the primers used in real-time PCR.

Gene	Forward Primer	Reverse Primer
**ACC**	CAACCACTACGGCATGACTCA	CGCAGAAGCAGCCCATTACTT
**CD 36**	TGAGCCTTCACTGTCTGTTGGAAC	AGGCTGTTGAGCACACCTTGAAC
**TNF-α**	GCATGATCCGAGATGTGGAACTGG	CGCCACGAGCAGGAATGAGAAG
**NF-κB**	TGTGGTGGAGGACTTGCTGAGG	AGTGCTGCCTTGCTGTTCTTGAG

## Appendix B

**Table A2:** The changes of weight.

Groups	0 Week	1th Week	2nd Week	3rd Week	4th Week
**ND**	199.51 ± 18.85 ^a^	227.56 ± 14.03 ^a^	246.26 ± 12.36 ^a^	241.92 ± 11.44 ^a^	229.05 ± 18.44 ^a^
**HFD**	205.14 ± 16.19 ^a^	239.49 ± 17.58 ^b^	271.30 ± 16.84 ^c^	262.30 ± 17.08 ^b^	252.04 ± 16.93 ^c^
**HLP-L**	199.85 ± 14.16 ^a^	240.26 ± 13.44 ^b^	261.65 ± 12.78 ^b^	261.57 ± 15.63 ^ab^	242.39 ± 16.95 ^b^
**HLP-H**	197.56 ± 13.97 ^a^	230.26 ± 13.39 ^a^	249.40 ± 17.03 ^a^	251.02 ± 17.79 ^ab^	241.77 ± 13.95 ^b^

Data are expressed as the mean ± SEM (*n* = 8). Values within a column with different letters are significantly different (*p* < 0.05).
